# The Usefulness of Intraoperative Indocyanine Green Fluorescence Imaging in Surgical Treatment of Refractory Chylothorax in Pediatric Patients: A Case Report

**DOI:** 10.70352/scrj.cr.24-0112

**Published:** 2025-02-18

**Authors:** Kanji Ishizu, Kanta Araki, Koji Kagisaki, Hideto Ozawa

**Affiliations:** Department of Pediatric Cardiovascular Surgery, Osaka City General Hospital, Osaka, Osaka, Japan

**Keywords:** chylothorax, indocyanine green, intra-operative fluorescence imaging, pediatric

## Abstract

**INTRODUCTION:**

Chylothorax is one of the complications in cardiovascular surgery. Although prolonged chylothorax leads to critical status and is associated with high mortality, its treatment has not been well established. We present a successful case of surgical treatment of chylothorax in a neonate using indocyanine green to identify the site of lymphatic leakage.

**CASE PRESENTATION:**

The patient with complete atrioventricular septal defect, patent ductus arteriosus, pulmonary hypertension, and chromosomal abnormality with trisomy 21 underwent pulmonary artery banding and patent ductus arteriosus ligation through median sternotomy. The postoperative course was complicated with chylothorax; conservative treatment was not effective, so surgical treatment was selected. Indocyanine green was injected subcutaneously between the first and second toes on the left side 30 min before surgery to identify the site of leakage. We could detect the lymphatic leakage from the para-aortic lymph node by indocyanine green camera in the left thoracic cavity, and the leakage sites could be closed with interrupted sutures.

**CONCLUSION:**

Identification of lymphatic leakage sites using indocyanine green could be an effective technique in the surgical treatment of chylothorax in pediatric patients.

## INTRODUCTION

Prolonged chylothorax can lead to critical conditions such as malnutrition, immunocompromised state, and fluctuating intravascular volume and is associated with a high mortality rate, especially in pediatrics.^1)^ Despite its importance, medical therapy is not well-established. Surgical treatment must be considered in refractory or long-standing cases. Intraoperative administration of cream or olive oil through nasogastric tubes is one of the options to determine the leakage points^2)^; however, because the lymphatic fluid is transparent and the leakage rate is very slow, it is difficult to identify the leakage sites and confirm whether the leakage has stopped after surgical closure. In adults, intraoperative indocyanine green lymphangiography has been reported to be effective in determining thoracic duct and lymphatic leakage.^3)^ In contrast, the effectiveness of indocyanine green in pediatric patients is still unclear.

We report a successful surgical treatment of refractory chylothorax using intraoperative fluorescence imaging with indocyanine green to identify leakage sites during the neonatal period.

## CASE PRESENTATION

**Fig. 1:**
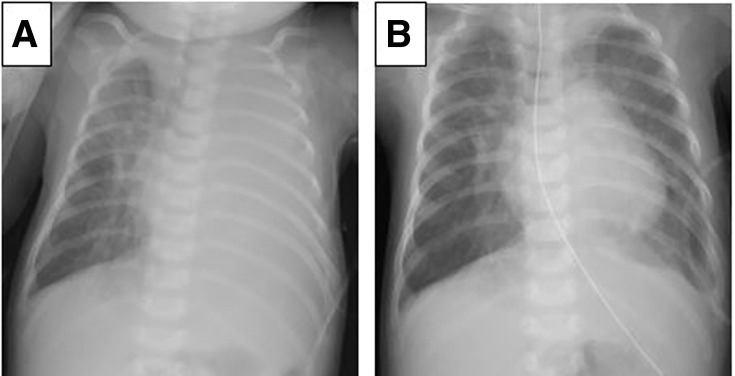
(**A**) Chest x-ray on post-operative day 16 showed unilateral pleural effusion. A diagnosis of chylothorax was made based on the biological examination of the pleural effusion. (**B**) The chest x-ray on discharge showed no pleural effusion on both sides.

The patient was born at 38 weeks’ gestation (2880 g) by vaginal delivery without any abnormalities during pregnancy. Complete atrioventricular septal defect, patent ductus arteriosus, and pulmonary hypertension were confirmed by transthoracic echocardiogram. Chromosomal abnormality with trisomy 21 was also noted. On postnatal day 20, pulmonary artery banding and patent ductus arteriosus ligation were performed through median sternotomy without any injuries to the pleura or thoracic duct. On postoperative day 16, tachypnea and retractions occurred suddenly. Chest x-ray showed left pleural effusion (**Fig. 1A**). We performed drainage of the left pleural cavity and milky fluid was drained, whose triglyceride value was 3067 mg/dL and lymphocyte fraction was 91.4%. The serum triglyceride was 165 mg/dL and the ratio of pleural fluid to serum triglyceride was 18.59. A diagnosis of chylothorax was made, and we started conservative therapy. Initially, we converted oral dietary to medium-chain triglyceride milk, and fibrogammin was administered for 5 days. Nonetheless, there was no tendency for improvement of chylothorax. Therefore, octreotide acetate was administered at a maximum dose of 200 µg/kg/day 4 days after diagnosis. However, the total drainage volume of pleural effusion remained around 20 mL/kg/day, and lymphatic vessel scintigraphy showed that the lymphatic fluid leakage was located on the left side of the pleural cavity.

**Fig. 2:**
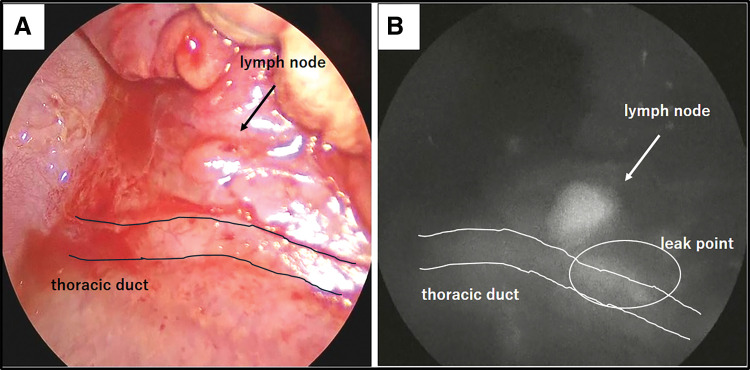
(**A**) The lymph nodes and leakage points could not be identified visibly in normal mode. (**B**) We could observe the thoracic duct and the para-aortic lymph node being stained with indocyanine green and the leakage point through indocyanine green mode at the same area.

We performed lymphatic fistula closure under general anesthesia in the right lateral decubitus position 18 days after the diagnosis. We injected 0.1 mL of 2.5 mg/mL indocyanine green subcutaneously between the left first and second toes 30 min before the skin incision. A left thoracotomy was performed through the 6th intercostal space. We detected lymphatic leakage from the para-aortic lymph node by the indocyanine green camera of Karl Storz (Tuttlingen, Germany) (**Fig. 2**). A total of 2 leakage sites were sutured with a total of 4 sutures using 6-0 polypropylene suture.

We started oral intake of medium-chain triglyceride milk on postoperative day 6, and the drainage volume did not increase. Therefore, we removed the left chest tube on postoperative day 13. The patient was discharged without recurrence of chylothorax or other complications (**Fig. 1B**).

## DISCUSSION

We identified 2 important clinical findings: First, we suspected chylothorax as the cause of sudden unilateral pleural effusion, although there was no direct injury to thoracic ducts or lymph nodes after cardiovascular surgery. It has been reported that thoracic ducts can be easily injured only by slight lymphatic congestion due to high central venous pressure.^4)^ In this case, it was suggested that the change in venous pressure caused by pulmonary artery banding resulted in thoracic duct injury. In addition, it has been pointed out that genetic disorders, including trisomy 21, are a risk factor for the development of chylothorax.^5)^ Therefore, we should suspect chylothorax as the cause of sudden pleural effusion even if no procedures were performed in the pleural cavity.

Second, indocyanine green was very effective in identifying leakage during surgical treatment in pediatric patients, even in neonates. Visualization of lymphatic vessels, including thoracic ducts, has been tried in the field of esophageal and pulmonary surgery in adults,^3)^ but it has rarely been tried in pediatric patients, especially in neonates. In this case, using indocyanine green to determine the leakage points in a neonatal case during the surgical procedure, we could confirm the leakage points and suture it without ligating the thoracic duct itself. This result indicated that the use of indocyanine green could also be an effective technique for the surgical treatment of chylothorax, even in the neonatal period.

## CONCLUSION

Identification of lymphatic leakage sites using indocyanine green could be a very effective technique in the surgical treatment for refractory and prolonged chylothorax after conservative therapies in pediatric patients and even in neonatal patients.

## DECLARATIONS

### Funding

No funding was received for this project.

### Authors’ contributions

Kanji Ishizu and Hideto Ozawa developed the theory.

Kanta Araki and Koji Kagisaki encouraged Kanji Ishizu to investigate and supervise the findings of this work.

All authors discussed the results and contributed to the final manuscript.

All authors read and approved the final manuscript.

All authors agreed to take responsibility for all aspects of the research.

### Availability of data and materials

The data that support the findings of this study are available from the corresponding author, Hideto Ozawa, upon reasonable request.

### Ethics approval and consent to participate

The Osaka City Hospital Institutional Review Board approved this case report (1902139), and opt-out consent was obtained in place of the individual written informed consent statement.

### Consent for publication

Informed consent to publish was obtained.

### Competing interests

The authors declare no conflicts of interest.
